# Modeling, optimization and efficient use of MMT K_10_ nanoclay for Pb (II) removal using RSM, ANN and GA

**DOI:** 10.1038/s41598-023-35709-0

**Published:** 2023-05-24

**Authors:** Farshad Hamidi, Abbas Norouzian Baghani, Mahboobeh Kasraee, Mehdi Salari, Mohammad Hadi Mehdinejad

**Affiliations:** 1 Department of Environmental Health Engineering, School of Public Health, Environmental Health Research Center, Golestan University of Medical Sciences, Gorgan, Iran; 2 Department of Environmental Health Engineering, School of Public Health, Tehran University of Medical Sciences, Tehran, Iran; 3 Department of Environmental Health Engineering, School of Public Health, Sabzevar University of Medical Sciences, Sabzevar, Iran

**Keywords:** Environmental chemistry, Pollution remediation

## Abstract

Regarding the long-term toxic effects of Pb (II) ions on human health and its bioaccumulation property, taking measures for its reduction in the environment is necessary. The MMT-K_10_ (montmorillonite-k_10_) nanoclay was characterized by XRD, XRF, BET, FESEM, and FTIR. The effects of pH, initial concentrations, reaction time, and adsorbent dosage were studied. The experimental design study was carried out with RSM-BBD method. Results prediction and optimization were investigated with RSM and artificial neural network (ANN)-genetic algorithm (GA) respectively. The RSM results showed that the experimental data followed the quadratic model with the highest regression coefficient value (R^2^ = 0.9903) and insignificant lack of fit (0.2426) showing the validity of the Quadratic model. The optimal adsorption conditions were obtained at pH 5.44, adsorbent = 0.98 g/l, concentration of Pb (II) ions = 25 mg/L, and reaction time = 68 min. Similar optimization results were observed by RSM and artificial neural network-genetic algorithm methods. The experimental data revealed that the process followed the Langmuir isotherm and the maximum adsorption capacity was 40.86 mg/g. Besides, the kinetic data indicated that the results fitted with the pseudo-second-order model. Hence, the MMT-K_10_ nanoclay can be a suitable adsorbent due to having a natural source, simple and inexpensive preparation, and high adsorption capacity.

## Introduction

Environmental pollutants such as heavy metals and their toxicity cause several problems around the world^1^. Heavy metals such as lead (Pb (II)) ions, cadmium (cd), nickel (Ni), and zinc (Zn) have been found in the effluents of various industries including battery manufacturing, color production, mining activities, refining and melting industries, radiators manufacturers and wood preservation^2–5^.

The United States Environmental Protection Agency (U.S. EPA) has determined an allowable limit of 0.1 mg/L for Pb (II) ions in surface water and 0.05 mg/L for drinking water^6^. Exposure to high levels of Pb (II) ions may cause disorders of hemoglobin biosynthesis and anemia, hypertension, kidney damage, abortion and neonatal failure, nervous system disorder, brain damage, and behavioral disorders in children^7,8^. For this reason, the disposal of these metals into the environment is a serious danger to humans and the ecosystem^7,8^.

For the removal of heavy metals, several technologies being used, including ion exchange, chemical precipitation, ultrafiltration, solvent extraction, reverse osmosis, and adsorption, among others. However, many of them cause issues like complex monitoring or control systems, expensive reagents and equipment, toxic sludge disposal, reduced removal efficiency, etc^9^. The adsorption process is one of the most successful methods for Pb (II) ions removal from polluted waters^10^. Various sorbents including chitosan, various leaves, barks, and peels, as well as inorganic elements like clay, zeolite, activated alumina, clarified sludge, red, fly ash, and rice husk ash have been used to remove Pb (II)^11^.

One of the most important adsorbents for removing Pb (II) ions from contaminated water is MMT-K_10_ (Montmorillonite-K_10_)^12^. The MMT-K_10_ belongs to a family of layered silicates called smectite or 1:2 layered silicates. Each layer is about one nanometre thick and consists of two SiO_4_^4−^ tetrahedral silica plates comprising an AlO_6_ octahedral aluminium hydroxide plate^13^. By placing the silicate layers on each other, a space is created between them known as the interlayer space. These layers are interconnected by weak Vander Waals force^13^. Aluminium substitution by magnesium or ferrous iron creates a negative charge, which is neutralized by cations such as sodium, potassium, or calcium in the interlayer space^13^. Given their properties such as the high layer charge, adsorption ability, high specific surface area, shrink-swell capacity, and colloidal properties, clay minerals have attracted the attention of many researchers as heavy metal adsorbents^14^.

Artificial Intelligence (AI) is a well-known discipline of computer science that is concerned with the development of intelligent systems and the resolution of problems in a manner similar to that of a human intelligence system^15^. AI systems are useful in practically all interdisciplinary domains, and they have been used in a variety of optimization, classification, regression, and forecasting applications. To improve the precision of optimum solution prediction, AI technologies are frequently used with experimental design approaches such as response surface methodology (RSM)^16^. Likewise, AI is predicted to save 20 to 30% of operating costs by lowering the cost of the chemical and improving its use in water treatment^16^. Because of its plain implementation, adaptability, generalization, and convenient design, AI applications in water treatment have made the procedure simple. Several pieces of research revealed the successful use of various AI techniques in the modeling and optimization of water treatment processes, such as the removal of contaminants from water.

Through prediction, diagnosis, assessment, and simulation, these approaches are widely utilized to deal with wastewater treatment operations, water reuse, water conservation, and cost reduction. Artificial neural networks (ANN), genetic algorithms (GA), decision trees (DT), fuzzy inference systems (ANFIS), and other AI techniques are extensively employed in water treatment^17^. Abolino et al. have examined the interaction of metal ions with the MMT-K_10_ and vermiculite minerals. Baraka et al. (2011), Bhattacharya and Gupta (2006), and Akpomie (2015) have reported that by decreasing the particle size and increasing the amount of clay (adsorbent), the contact area and the rate of adsorption increased^18–20^.

The novelty of this work is determining the morphology of the adsorbent and examining the Pb (II) ions removal by MMT-K_10_ nanoclay adsorbent from aqueous solutions and the optimizing of the adsorption process through different methods. Furthermore, many studies have focused on genetic algorithm with neural network hybrid technique (ANN-GA) of heavy metals removal from aqueous solution using Walnut Shell^9^, Rice husk^2,21^, Arachis hypogaea’s shell^22^, pumice^23^ and rice husk char^24^, Few studies have assessed concurrently the comparison of three common optimization methods in the absorption process including the response surface method (RSM), genetic algorithm (GA), and artificial neural network (ANN), and the degree of similarity of the obtained results was investigated and in adequate information is available regarding this comparison methods. Finally, we have investigated the morphology of the adsorbent and examined the Pb (II) ions removal by MMT-K_10_ nanoclay adsorbent from aqueous solutions regarding the adsorption process including RSM and ANN-GA.

Hence, this study was conducted to (1) investigate the morphology of the MMT-K_10_ nanoclay adsorbent, (2) examine the Pb (II) ions removal by MMT-K_10_ nanoclay adsorbent from aqueous solutions, (3) compare three common optimization methods in the adsorption process (i.e., RSM and ANN-GA) and (4) investigate the degree of similarity between the obtained results.

## Materials and methods

### Materials

All chemicals, including sulfuric acid (H_2_SO_4_, 98%), sodium hydroxide (NaOH, ≥ 97.0%), and tetrazolium solution of Pb (II) ions (1000 mg/L) with 99% purity were purchased from Merck Co, (Germany). The montmorillonite-K10 nanoclay was purchased from Sigma Aldrich Company. Pb (II) ions concentrations measurement was performed using Atomic Absorption Spectrometer (YOUNGLIN, Model AAS 8020). The laboratory pH meter (Metrohm model 827 pH meter) was used for the pH determination.

### Morphology of MMT-K_10_ nanoclay

The morphology of the adsorbent was characterized by Scanning Electron Microscope (SEM and EDAX) (TeScan—Mira III Czech Republic), Fourier Transform Infrared spectroscopy (FTIR) (PerkinElmer Spectrum RX II, USA). The crystalline phase was detected by X-Ray diffraction (XRD) (Philips Pert, MPD model) operated with cobalt tube at 40 mv and 40 mA in the range of 2θ from 5° to 80°. The Brunauer–Emmett–Teller (BET) for determining of chemical properties of the MMT-K_10_ and X-ray Fluorescence (XRF) for determining of MMT-K_10_ chemical components was used.

### Batch adsorption studies

Initially, different concentrations of Pb (II) were prepared from stock solution.The experiments were conducted in 100 ml Erlenmeyer flasks with each flask containing 25 ml of the desired concentration of Pb (II) solution. The pH of each flask was then adjusted with a solution of NaOH and HCL 0.1 N solution, and samples were shaken for a predetermined amount of time at room temperature. The samples were run through a 0.45 μm Whatman filter prior to the detection of Pb (II) ions. Atomic Absorption Spectrometer read the final concentration of Pb (II). The mass balance equation, was used to determine the amount of adsorbed Pb (II).

$${\mathrm{q}}_{\rm{eq}}=\frac{({\mathrm{C}}_{^\circ }-{\mathrm{C}}_{\rm{t}})\mathrm{V}}{\mathrm{M}}$$

In which C_0_ and C_e_ (mg/l) are initial and residual Pb (II) concentration, V (L) is the Pb (II) solution volume and m (g) is the adsorbent mass.

### Box–Behnken design optimization of parameters

Box–Behnken design (BBD) is chosen as a particular kind of response surface methodology (RSM). In this work, RSM-BBD was applied for statistical analysis of the experimental data using the Design-Expert software version 11. The associations between the removal of Pb (II) ions by MMT-K_10_ (as a response) and four operating parameters including pH (X1), adsorbent dosage (X2 (g/L)), reaction time (X3 (minute)), and Pb (II) ions concentration (X4 (mg/L)) were investigated (Table S1). Please refer to the Supporting Information for more specific details. The design of RSM-BBD is shown in Table 1. The following quadratic model (Eq. 1) can predict the removal efficiency of Pb (II) ions (Y%). The common quadratic model is exhibited by the following Eq. (1).

1 $$\mathrm{Y}= {\upbeta }_{0}+ \sum_{\rm{i}=1}^{\mathrm{n}}{\upbeta }_{\rm{i }}{\mathrm{x}}_{\rm{i}}+ \sum_{\rm{i}=1}^{\mathrm{n}-1}\sum_{\rm{j}=\mathrm{i}+1}^{\mathrm{n}}{\upbeta }_{\rm{ij}}{\mathrm{x}}_{\rm{i}}{\mathrm{x}}_{\rm{j }}+ \sum_{\rm{i}=1}^{\mathrm{n}}{\upbeta }_{\rm{ii}}{\mathrm{x}}_{\rm{i}}^{2}$$

where Y is the predicted amount of Pb (II) ions removal (mg/L). Xi and Xij are the non-coded parameters and n is the number of factors. β0, βi, βii, and βij are the model coefficients.

**Table 1:** BBD design and the response of each run and predictions.

Run	A: pH (–)	B: MMT ( g/L)	C: time (minute)	D: Pb (II) ions (mg/L)	Ce ( mg/L)	RE (%)	RSM predict (%)	ANN predict (%)
1	3	0.55	105	50	12.39	75.22	74.81	75.16
2	5	0.55	105	30	2.16	92.8	92.38	92.42
3	3	0.55	180	30	6.81	77.3	77.54	77.19
4	7	1	105	30	3.186	89.38	89.46	90.44
5	3	1	105	30	5.31	82.3	82.38	82.31
6	5	0.55	105	30	2.25	92.5	92.38	92.42
7	5	0.55	105	30	2.4	92	92.38	92.42
8	7	0.55	180	30	4.74	84.2	84.13	84.10
9	3	0.55	30	30	6.06	79.8	80.14	79.57
10	5	0.55	30	10	0.92	90.8	91.01	90.63
11	7	0.55	30	30	3.864	87.12	87.16	87.12
12	5	0.55	30	50	6.64	86.72	86.52	86.64
13	5	1	105	50	5.51	88.98	89.13	88.92
14	5	0.55	180	50	8.065	83.87	83.41	83.80
15	5	0.1	180	30	3.465	88.45	88.41	95.64
16	7	0.55	105	10	1.4	86	86.40	86.00
17	5	0.1	105	50	7.85	84.3	85.33	84.27
18	5	0.1	30	30	2.61	91.3	90.89	91.09
19	7	0.55	105	50	8.94	82.12	82.01	82.11
20	5	0.55	105	30	2.34	92.2	92.38	92.42
21	7	0.1	105	30	3.99	86.7	86.36	86.59
22	3	0.55	105	10	2.01	79.9	79.99	78.38
23	3	0.1	105	30	5.95	80.17	79.83	80.11
24	5	1	30	30	1.794	94.02	94.05	93.96
25	5	1	105	10	0.63	93.7	92.94	93.71
26	5	0.55	180	10	1.145	88.55	88.49	89.69
27	5	1	180	30	2.85	90.5	90.90	90.44
28	5	0.1	105	10	0.903	90.97	91.09	91.94

### Isotherm and kinetic studies

Isotherm and Kinetic models that were used for the adsorption of Pb (II) ions onto MMT-K_10_ nanoclay are shown in Table S2. Please refer to the Supporting Information for more specific details.

### Optimization of the adsorption process

In modeling studies, after studying the effects of factors on the response variable and investigating the adequacy of the model, process optimization is necessary and inevitable. In this work, process optimization was considered with the aim of maximizing the removal of Pb (II) ions. In this study, different methods for removal optimization consisting of Design Expert Software (Numerical Section), Genetic Algorithm (GA), and artificial neural network (ANN) were investigated.

The GA approach is a class of numerical and combinational optimizers that are mainly helpful for solving complex non-linear and non-convex issues. The GA approach has growingly been used in engineering in the past decade, owing to the GA approach being regarded as a tool for optimization in engineering design.

ANN approach is widely utilized to understand and predict complex system behaviors and has a promising ability in learning and classification of data. The enhancement of ANNs by using optimization approaches can remove some of their drawbacks in picking the best network structure using the proper optimization approaches. In the current study, a multi-layer perceptron ANN model with Levenberg–Marquardt back-propagation algorithm was established based on feed forward neural network architecture.

The feed forward neural network architecture was made of one input layer with four neurons including the independent variables, one hidden layer, and one output layer with one neuron (adsorption efficiency for Pb (II) ions removal). The number of neurons in the hidden layer was optimized based on the largest value of R^2^ and the lowest value of mean squared error (MSE) between experimental and predicted values. Sigmoid transfer function and pureline transfer function were applied for the hidden layer and for the output layer, receptively. The experimental design matrix represented in Table 1 was divided randomly into three sets including training (70%), validation (15%), and test (15%) data. Since the difference in the dimensions and the range of the input variables may cause computational problems to address this issue, normalization of the data was done in the range of 0.1– 0.9 by the following Eq. (2).

2 $${\mathrm{Y}}_{\rm{i}}=0.1+0.8 \times \frac{{\mathrm{x}}_{\rm{i}}- {\mathrm{x}}_{\rm{min}}}{{\mathrm{x}}_{\rm{max}}- {\mathrm{x}}_{\rm{min}}}$$

where, Y_i_ is the normalized X_i_, and X_max_ and X_min_ stand for the maximum and minimum level of variable x_i_, respectively. The ANN model was implemented in MATLAB 2013a software.

## Results and discussion

### Morphology of MMT-K_10_

#### BET

The BET specifications of MMT-K_10_ are given in Table S3. The results of BET experiment showed that the specific surface area, the size of nanoparticles, and the empty space of MMT-K_10_ were 220–270 m^2^/g, 1–2 nm, and 60 angstroms (Å), respectively.

#### XRF

The results of the chemical analysis of MMT-K_10_ components and elemental analysis by X-ray Fluorescence (XRF) are shown in Table S4. As can be seen from Table S4, the major constituents of MMT-K_10_ were silicate (SiO_2_) (50.96%) and aluminium oxide (Al_2_O_3_) (19.6%). The adsorption analysis indicates that the main constituents are inorganic materials. The distribution of the nanoclay in the polymer matrix can be determined by examining the distance between the crystalline layers.

#### XRD

To determine the chemical composition of the nanoparticles, X-ray diffraction pattern (XRD) made by Philips X’Pert MPD model with cobalt tube at 40 mv and 40 mA was used. XRD analysis on MMT-K_10_ nanoparticles is shown in Fig. S1. The presence of a strong peak in the sample XRD pattern indicates an interlayer spacing in MMT-K_10_. The XRD results showed that MMT-K10 is hexacoordinated in the solid state. Considering that chemical formula of nanoparticles of MMT-K10 is (Na,Ca)0.3(Al,Mg)2(Si4O10)(OH)2·nH2O^25^. X-Ray diffraction was used to find out the nature of the adsorbent and it was in agreement with the standard pattern data (JCPDS74-1811). According to Fig. S1, the observed peak at 2θ = 23.7 can be related to cristobalite^26^. The observed peaks at 2θ = 19.82, 20.9, 26.68, 27.77, 50.11, and 59.97 can be related to the presence of quartz as the main constituent of MMT-K_10_^26–31^.

#### Field emission scanning electron microscopy (FE-SEM) with energy dispersive X-ray spectroscopy (EDS)(FE-SEM & EDS)

**Figure 1:**
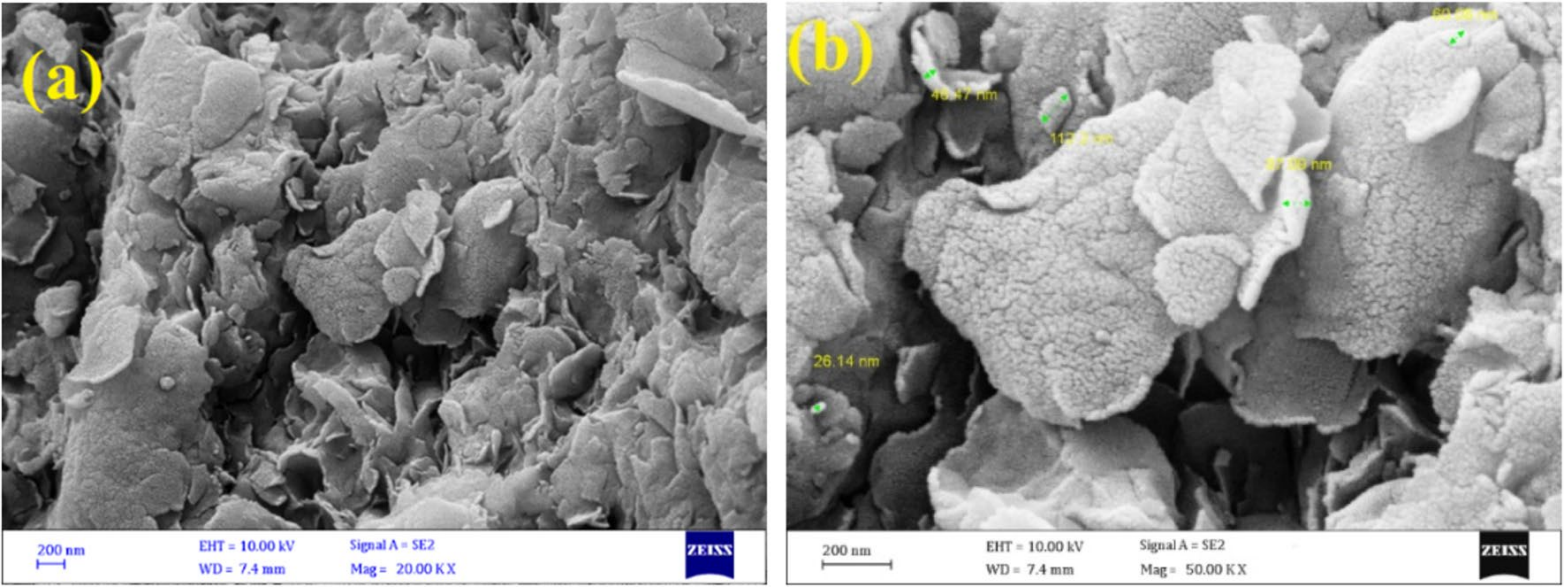
FE-SEM of MMT-K_10_.

The surface morphology of the adsorbent is presented as FE-SEM in Fig. 1. According to Fig. 1, MMT-K_10_ has a flaky, multilayer structure with interlayer spaces between its layers, and each layer is formed by agglomerated nanoparticles in the range of 26 to 113 nm. The EDS analysis indicates the elemental analysis of MMT- K_10_. As it is evident in Fig.S2, the constituent component of MMT- K_10_ was the mineral phase. The constituent components of MMT- K_10_ were “O”, Si, Al, Fe, Ca, Mg, and Na with a percentage of 42.3%, 31.4%, 14.2%, 8.2%, 1.7%, 1.4%, and 0.8%, respectively.

#### FTIR

FTIR spectra of MMT-K_10_ are demonstrated in Fig. S3. Accordingly, the adsorption occurred at 467 and 531 cm^−1^ can be related to Si–O–Mg and Si–O–Al bending vibrations, respectively^30^. Two peaks at 696 and 789 cm^−1^ asserted the presence of Si–O band^26,27^. The Si–O stretching vibration appeared at the peak 1034 cm^−1^^26–32^. An observed peak around 3418 was described to the stretching vibration of the OH groups, revealing the adsorption of water on MMT-K10 surface^18,27,30–32^.

### Statistical analysis

#### RSM based on BBD experimental design

Based on the BBD method, 28 tests were determined to check the efficiency of Pb (II) removal using MMT-K_10_ adsorbent. BBD design and the response of each run and prediction are shown in Table 1.

#### Analysis of variance (ANOVA) for response surface quadratic model

Analysis of variance for removal of Pb (II) ions by MMT-K_10_ is presented in Table 2. Based on Table 2, the highest F-value and the lowest p-value (less than 0.0001) for parameters indicate of the greatest effect of that parameter on the absorption process. According to Table 2, pH has the highest F-value (524.31) and the lowest p-value (less than 0.0001), which reflects the strong effect of pH on the adsorption of Pb (II) ions compared with other parameters. In addition, according to Table S5, among all studied models for removing Pb (II) ions by MMT-K_10_, a quadratic model was selected with the highest regression coefficient value (R^2^ = 0.9903) and insignificant lack of fit (0.2426). The highest regression coefficient value (R^2^ = 0.9903) and insignificant lack of fit (0.2426) indicated the validity of the quadratic model (Table S5).

**Table 2:** Analysis of variance for removal of Pb (II) ions by MMT-K_10_.

Source	Sum of squares	Df	Mean square	F-value	p-value	
Model	734.85	14	52.49	198.10	< 0.0001	Significant
A-pH	138.92	1	138.92	524.31	< 0.0001	*
B- MMT-K_10_	24.06	1	24.06	90.79	< 0.0001	*
C-Time	23.77	1	23.77	89.72	< 0.0001	*
D-Pb	68.69	1	68.69	259.24	< 0.0001	*
AB	0.0756	1	0.0756	0.2854	0.6022	
AC	0.0441	1	0.0441	0.1664	0.6899	
AD	0.1600	1	0.1600	0.6039	0.4510	
BC	0.1122	1	0.1122	0.4235	0.5265	
BD	0.9506	1	0.9506	3.59	0.0807	
CD	0.0900	1	0.0900	0.3397	0.5700	
A^2^	417.67	1	417.67	1576.31	< 0.0001	
B^2^	1.36	1	1.36	5.15	0.0410	
C^2^	19.24	1	19.24	72.62	< 0.0001	
D^2^	62.53	1	62.53	236.00	< 0.0001	
Residual	3.44	13	0.2650			
Lack of fit	3.08	10	0.3077	2.51	0.2426	Not significant
Pure error	0.3675	3	0.1225			
Cor total	738.29	27				

Moreover, the quadratic model for parameters affecting Pb (II) ions removal (Eq. 3), could be obtained in terms of real factors.

3 $${\text{RE}}\, = \,{92}.{38}\, + \,{3}.{4}0{\text{ A}}\, + \,{1}.{\text{42 B}} - {1}.{\text{41 C}} - {2}.{\text{39 D}} - {8}.{\text{34 A}}^{{2}} \, + \,0.{\text{4767 B}}^{{2}} - {1}.{\text{79 C}}^{{2}} - {3}.{\text{23 D}}^{{2}}$$

Where RE is the removal efficiency of Pb (II) ions (%). A, B, C, and D are pH, adsorbent dose, reaction time, and concentration of Pb (II) ions, respectively.

#### Confirming the mathematical model

Confirming the quadratic mathematical model by predicted vs. actual and residuals vs. predicted for Pb (II) ions removal in experimental runs is exhibited in Fig. S4. According to Fig. S4a, there is a tendency close to a straight line, which indicates a very good model for the prediction of the response variable. Fig. S4b shows that the obtained data set difference of predicted and residual values are between 2 and −2 and it can be concluded that the difference between these two values is small.

### The effect of studied variables on Pb (II) ions removal

#### The effect of pH

**Figure 2:**
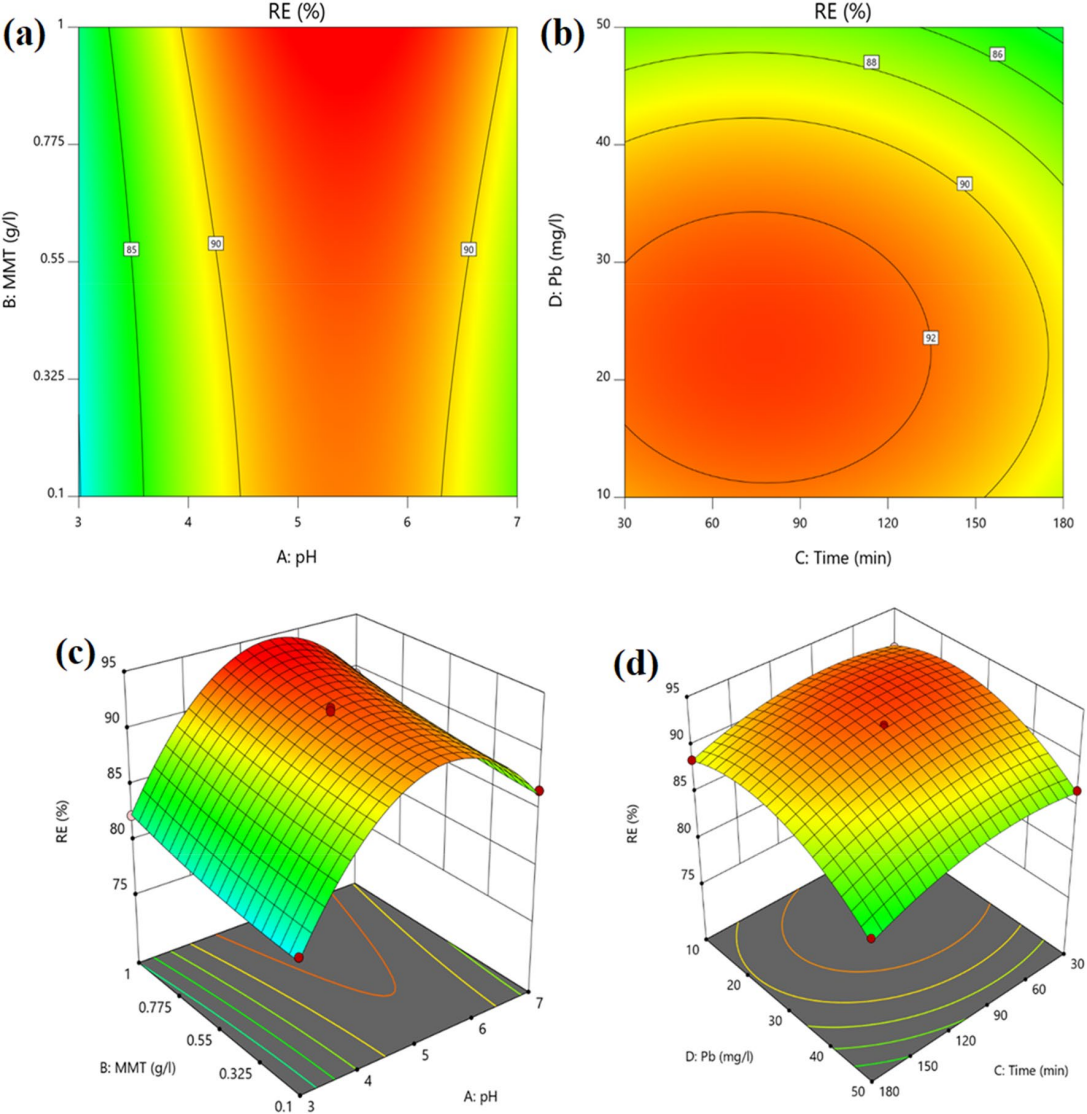
An interaction effect of variables on the adsorption of Pb (II) ions onto 2D & 3D contour MMT-K_10_: pH and adsorbent dose (**a,c**); initial concentration of Pb and contact time (**b,d**).

The effect of pH, reaction time, the dose of adsorbent, and initial concentration of pollutant on the adsorption of Pb (II) ions onto MMT-K_10_ is drawn in Fig. 2 (2-D and 3-D plots). The results show that the highest and lowest percentage of Pb (II) ions removal by MMT-K_10_ adsorbent were at pH  5 (equal to 89.5%) and pH  3 (equals 48%), respectively (Fig. 2). Considering that the highest percentage of lead ion removal occurs in pH  5 that pH is considered as the optimum pH. The pH of the solution affects changes in both the surface charge of MMT-K10 and the rate of ionization of the adsorbed molecules. When the pH increases and as we go towards alkaline mediums, the adsorption strength of the nanoparticles decreases. In contrast, the removal efficiency of the adsorbent in acidic mediums increases. The Pb (II) ions adsorption is largely dependent on the protonation or non-protonation of the amine or carboxylic groups present in the MMT-K_10_ nanoparticles^33,34^. By decreasing the pH of the solution, the amine groups present in the nanoparticle composition become protonated with varying degrees, hence reducing the number of available sites for chelating metal ions, resulting in electrostatic repulsion of metal cations^35^. However at higher pH, the ligands present in the adsorbent, such as the carboxylic group, increase the negative charge density on the surface of the ligands and the adsorption percentage will be increased. The optimum pH value for the heavy metal removal was obtained at 5, which causes the high efficiency of adsorption in acidic mediums, where in this case, the Pb (II) ions are well adsorbed on the specified adsorbent bands with H^+^.

#### The effect of contact time

The reaction time is one of the most important parameters in the adsorption process^36–38^. As can be seen in Fig. 2b, the optimal reaction time was obtained at 90 min on the adsorption of Pb (II) ions onto MMT-K_10_. According to Fig. 2b, the removal efficiency of Pb (II) ions increased from 79.8 to 94.9% with increasing the reaction time from 30 to 120 min. However, by increasing the reaction time from 120 to 180 min, the removal efficiency decreased from 94.9 to 83.9%. This could be attributed to the fact that the removal of Pb (II) ions by adsorbent was quick at the onset of the process of adsorption due to the availability of more adsorption sites, which is similar to previous works^36–40^.

#### The effect of concentration of Pb (II) ions

According to Fig. 2c, the highest removal of Pb (II) ions at a concentration of 30 mg/L was 93.46%. However, the lowest removal of Pb (II) ions at a concentration of 50 mg/L was 71.4%. Considering that the highest percentage of lead ion removal occurs in 30 mg/L, the concentration of 30 mg/L is considered as the optimum Pb (II) ions concentration. With the increase in the initial concentration from 30 to 50 mg/L the removal efficiency was increased from 93.5 to 71.4%. The results of this work indicated that as the initial concentration increases the adsorption efficiency decreases. The increase in the initial concentration of Pb (II) ions the gradient driving force of the concentration and the adsorption capacity was increased. The active sites on the adsorbent are highly available at low concentrations of Pb (II) ions. However, at higher concentrations of Pb (II) ions, the available sites on the adsorbent are limited and as a result, the absorption capacity decrease, which is consistent with the results of a previous study^41,42^. This could be attributed to the fact that the limitations in pore sizes and the increasing electrostatic repulsion force between the charges of Pb (II) ions gradually can be reduced the adsorption rate. Our findings were according to the obtained results by Jamali Armandi^4^.

#### The effect of adsorbent dose

According to Fig. 2d, the highest removal of Pb (II) ions at adsorbent dose of 1 g was 94%. However, the lowest removal of Pb (II) ions at adsorbent dose of 0.1 g was 79.83%. With the increase in the adsorbent dose from 0.1 to 1 g the removal efficiency was increased from 79.8 to 94%, respectively. It can be concluded that with the increase in the adsorbent mass, the specific surface area increases, which leads to easy absorption of Pb (II) ions onto MMT-K_10_.

#### The interaction effects of variables

An interaction effect of variables on the adsorption of Pb (II) ions on MMT-K_10_ is described in Fig. 2. According to Fig. 2a, with an increase in the adsorbent dose from 4.5 to 6 and adsorbent dose from 0.1 to 1 g/L, the uptake rate was increased from 80 to 95%, respectively.

As can be seen in Fig. 2b, by increasing in the concentration of Pb (II) ions from 10 to 30 mg/L and reaction time from 30 to 120 min, the uptake rate was increased from 87 to 95%, respectively.

### Mechanism of Pb (II) adsorption

Surface adsorption, partition, surface precipitation, and structural incorporation are the key associated mechanisms for uptaking pollutants^43–46^. Surface adsorption, which includes physical adsorption (van der Waals forces) and chemisorption (involving the formation of chemical bonds) is the concentration of pollutants on near the surface or pores of an adsorbent. Chemisorption is constantly involved in the adsorption of heavy metal cations and oxyanions on metal (hydr)oxides. The primary means of interaction between the adsorbed ions and the adsorbent is electrostatic interaction. Additionally, the "ion-exchange" process refers to the exchange of ionic pollutants with the pre-adsorbed ions on the adsorbent and adsorption of heavy metal cations is largely an ion-exchange controlled process. Surface-precipitation is the formation of precipitates on the adsorbent surface, which often requires relatively large concentrations of cations and anions. The adsorbents first concentrate pollutants on their surface through adsorption/ion-exchange, after which precipitates form because of an excess of cations and anions on the surface. The co-adsorption of cations (such as Cd^2+^, Pb^2+^) and oxyanions (such as phosphate, arsenate) on the surface of metal (hydr)oxides is a common adsorption process that involves surface-precipitation. According to O'Day and Vlassopoulos^45^, "structural incorporation" refers to the incorporation of ions into the solid phase of adsorbent, such as the sequestration of metal cations into the crystal structure of minerals through isomorphous substitution. This adsorption procedure often has a poor adsorption rate and invariably comes after the surface adsorption procedure. This kind of adsorption can effectively sequester the pollutants as they are integrated into the bulk phase of the adsorbents^44–47^. Nonetheless, it should be noted that different literatures might classify adsorption mechanisms differently. Moreover, the adsorption of pollutants onto adsorbents frequently involves multiple types of processes. For instance, the simultaneous intake of heavy metal cations and metal (hydr)oxides involves structural incorporation, surface precipitation, and surface adsorption. For Pb (II) adsorption removal can like this (1) the filling of MMT pores by Pb(II) ions, (2) the functional groups present in the MMT have interaction with the Pb(II) ions, and (3) the complex formation in between the Pb(II) ions and the functional groups^11^.

### Isotherm and kinetics studies

Adsorption isotherms, in general, offer crucial information for maximizing the utilization of adsorbents. It is possible to obtain descriptions of sorbate and sorbent affinity, bond energy, and adsorption capacity, to name a few, from isotherm equilibrium models that apply to adsorption processes^48^. Figure S5 depicts the non-linear isotherm and non-linear kinetic models of Pb (II) ions removal by utilizing MMT-K10. The interaction between Pb (II) ions and the adsorbent was assessed using the isotherm models (Fig. S5a). Table 3 displays the isotherm model parameters for the Pb (II) ion adsorption process using MMT-K10. With a correlation coefficient of 0.983, Table 3 shows that the adsorption of Pb (II) ions is more consistent with the Langmuir isotherm model. The single layer of Pb (II) ions adsorption on MMT-K10 is confirmed by the experimental results, which shows that the Langmuir isotherm is a better fit to the data. The Langmuir adsorption model is based on monolayer sorption occurring on a homogenous surface without interaction between sorbed species. According to the Langmuir model, chemical interaction forces and adsorptive forces are similar^48^. Studies conducted by Susmita Sen Gupta et al.^49^, SM Dal Bosco et al.^50^, Ali Sdiri et al.^51^, Dong-Su Kim^48^, Carvalho et al.^52^ showed similar results. Furthermore, the maximum sorption capacity of Pb (II) ions adsorption on MMT-K10 was 40.86 mg/g and achieved at pH  5. The high adsorption capacity of MMT-K10 compared to other adsorbents such as Coconut coir pith activated carbon (22.8 mg/g)^53^, Ukrainian chamotte clay (11 mg/g)^54^ and Kazakhstani natural zeolite (14 mg/g)^54^ presents a good potential of MMT-K10 nanoclay for Pb (II) ions removal. In addition, the Langmuir K_L_ constant is a measure of the metal ions affinity to the adsorption sites. Hence, the higher value of K shows a better adsorption. The value of K_L_ (L/mg) (Langmuir constant) for this work was 0.58. It shows that the adsorption process reached the equilibrium point in 0.58 L/mg of adsorbent surface. The affinity to the adsorption sites between Pb (II) ions and MMT-K10 was determined by separation factor (R_L_) (Eq. 10 in Table S2). The RL value above one describes an unfavorable adsorption while the RL values among 0 to 1 describe a favorable reaction. RL value of zero mentions at an irreversible reaction while RL value of one describes a linear reaction^55^. In present work, the value of computed RL was less than one, which means, the adsorption of Pb (II) on MMT-K_10_ is a favorable process.

**Table 3:** The parameters of isotherm and kinetic models for Pb (II) ions using MMT-K_10_.

The parameters of isotherm models for Pb (II) ions using MMT-K_10_								
Non-linear Freundlich			Non-linear Langmuir					
R^2^	K_F_ (mg/g)	n (L/g)	R^2^	q_m_ (mg/g)	K_L_ (L/mg)			
0.8275	16.656	3.242	0.9839	40.86	0.5813			

Non-linear Temkin			Non-linear Dubinin–Radushkevich					
R^2^	K_T_ (L/g)	B_1_	R^2^	K_D_	q_s_			
0.882	5.640	286.781	0.9162	0.001	35.91			

The parameters of kinetic models for Pb (II) ions using MMT-K_10_								
Pseudo-first-order (PFO)			Pseudo-second-order (PSO)			Intraparticle diffusion		
R^2^	q_e_ (mg/g)	K_1_ (min^−1^)	R^2^	q_e_ (mg/g)	k_2_ (g/mg) (min^−1^)	R^2^	C	K (mg/g min^1/2^)
0.954	2.015	0.078	0.981	2.796	0.023	0.931	−0.022	0.346

Kinetic models based on experimental data can provide vital details regarding the adsorption mechanism and rate-controlling stage, in addition to illustrating the relationship between the contact time and the amount of pb(II) absorbed by the adsorbent^56^. Adsorption mechanisms can be studied using a variety of adsorption kinetic models. Models for intraparticle diffusion kinetics, pseudo-first order, and pseudo-second order were all used in this work. The parameters of kinetic models for Pb (II) ions using MMT-K10 are shown in Table 3. As shown in Table 3, the equilibrium data were fitted onto three kinetic models: pseudo-first-order (PFO), pseudo-second-order (PSO) and Intraparticle diffusion. The PSO model had a higher correlation coefficient (R^2^ = 0.981) than the PFO model (R^2^ = 0.954) and intraparticle diffusion. The values of K_1_ and K_2_ were calculated from the the non-linear plot of qe vs. t. The value of k_2_ achieved in this investigation was 0.023 (g/(mg min)), and when compared to a study on magnetic bentonite, which has a similar structure to this adsorbent and had a value of 0.027 (g/(mg min)), the results were similar^57^. According to Gupta et al.^49^ the pseudo-second-order kinetic model fits the kinetics of the Pb(II) adsorption kaolinite and montmorillonite quite well. Pb(II) adsorption by zeolite materials of Municipal solid waste incineration fly ash (MSWI) follows the pseudo-second-order kinetic model, according to Qili Qiu et al^58^. According to. Guerra et al., both natural and functionalized Brazilian bentonite's Pb(II) adsorption kinetics follow the pseudo-second-order model^59^. The pseudo-second-order rate equation can be used to explain the kinetics of Pb(II) adsorption by magnetic bentonite, as Chenglong Zou et al.^57^ demonstrated. Considering above-mentioned studies, the pseudo-second-order model was mostly found to be well fitted to describe kinetic of Pb(II) adsorption from aqueous solutions.

### Disposal of the adsorbent into the environment

Adsorbents must first be properly disposed or recycled before they can be employed extensively in pollution management. Several studies addressed this problem and produced several methods for dealing with the used adsorbents, which will be briefly discussed in this part. The Pb-loaded MMT disposal is not recommended in the open environment as it may leach^2^. Because of its economical cost and great efficacy, MMT is arguably the best choice in this group for the removal of cationic pollutants (such as heavy metal cations, radioactive nuclides, and cationic dyes)^47^. After the adsorption of radioactive nuclides (such as La^3+^, and UO_2_^2+^) and heavy metal cations (such as Pb^2+^,Cd^2+^, Cu^2+^), one method for disposing of spent Materials safely involves in-situ sequestering the adsorbed pollutants by heat treatment^47,60–62^. In other words, heating the used MMT at a very high temperature can make the interlayers collapse, which will then block the desorption pathway for the pollutants that have been adsorbed, resulting in in-situ sequestration of contaminants inside the MMT interlayers. As a result, the sequestration efficiency of the pollutants that have been adsorbed often improves with heating temperature^47,60–63^. Even at relatively moderate heating temperatures, some heavy metal cations with small ionic radius, such as Ni^2+^ and Cu^2+^ can move into MMT layers. (for instance, below 300 °C).

### Optimization

#### Software-numerical method (or RSM)

The optimal reaction conditions are the value of each variable in which the maximum uptake rate is obtained. In this work, optimal reaction conditions were determined through the numerical optimization method. According to the RSM, optimal reaction conditions were pH  5.45 pH, adsorbent dose = 0.98 g/L, the concentration of Pb (II) = 25 mg/L, and reaction time = 68 min, for maximum efficiency of 95.149%. Optimal condition was investigated, in which the removal efficiency of Pb (II) was 93.1%.

#### GA method

**Figure 3:**
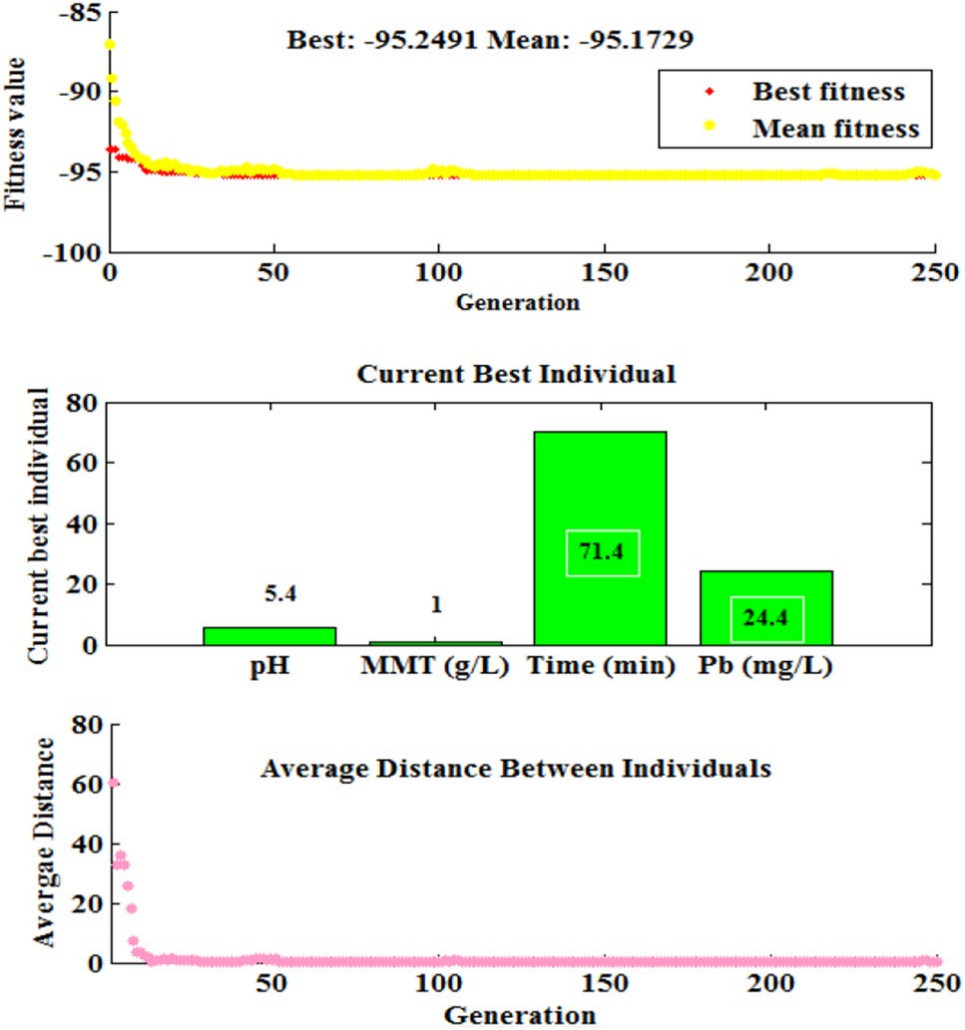
The genetic algorithm (GA) optimization of the removal of Pb (II) using MMT-K_10_.

In addition to the software-numerical section, the proposed approach was performed in Matlab GA toolbox as genetic algorithm (GA) optimization for the removal of Pb (II) using MMT-K_10_. To optimize the RSM-CCD model based on the GA approach, the minimum and maximum levels of the independent variables were set at the upper and lower levels. As seen in Fig. 3, the results showed the best fitness value was improved rapidly until about generation 50. After that, the results showed no impressive improvement and were almost constant since populations got closer to the optimal point. As shown in Fig. 3, the maximum removal efficiency (95.2%) was achieved at the optimum conditions. Hence, according to the GA method, optimal reaction conditions were 5.4 pH, 1 g/L adsorbent, 24.4 mg/L concentration of Pb (II), and 71.4 min of the reaction time, for a maximum efficiency of 95.2%.

#### ANN model

In the present work, the ANN model was used for developing a mathematical model based on the findings of experimental design in Table 1.

**Figure 4:**
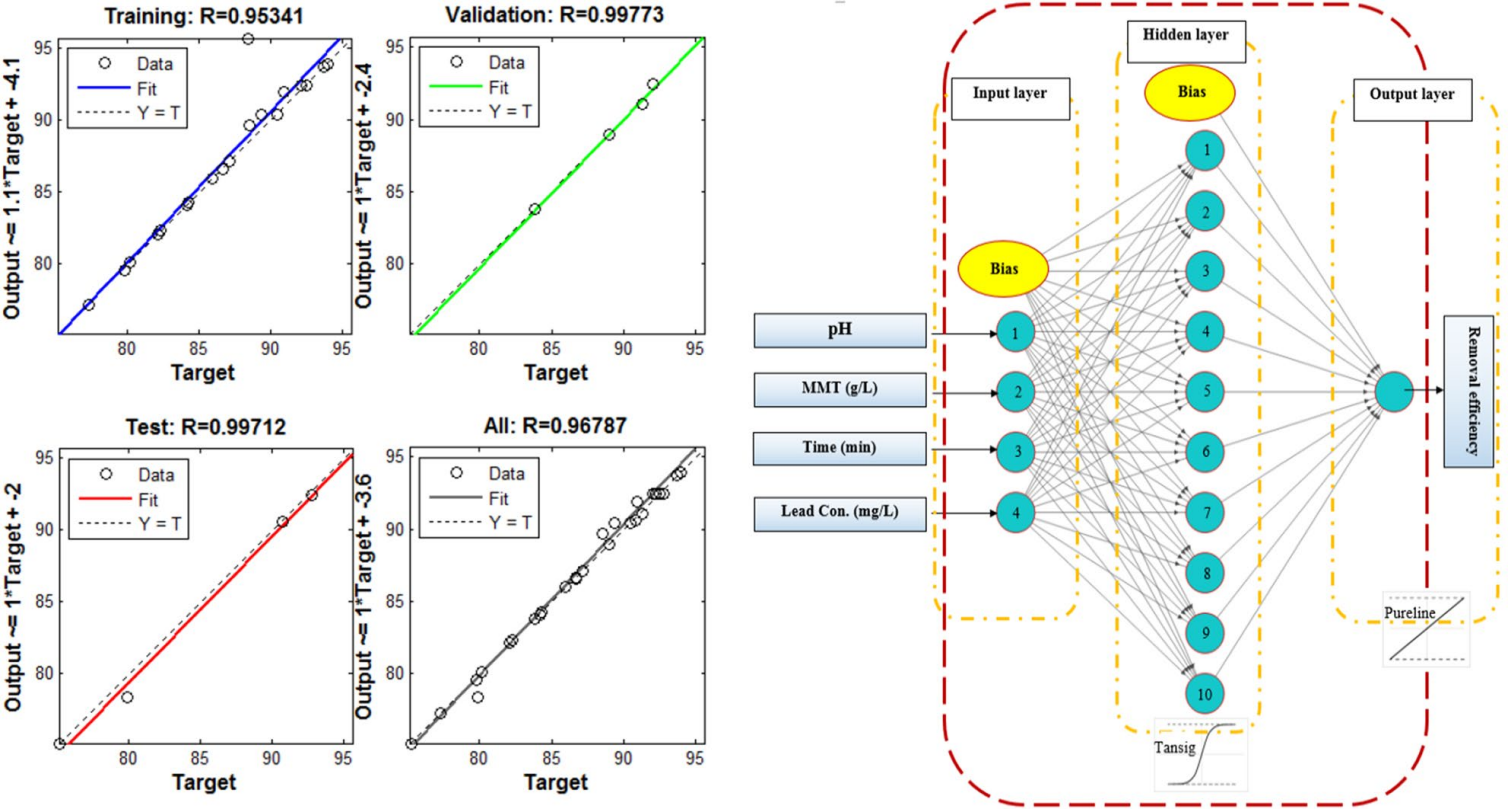
The ANN model for optimization of the removal of Pb (II) ions using MMT-K_10_.

The independent variables and experimental removal efficiency serve as the inputs and outputs of the ANN model, respectively. According to Fig. 4, the architecture of the neural network consists of four neurons in the input layer, while one neuron makes up the output layer. The optimized number of neurons in the hidden layer was explored by changing the number of neurons in the hidden layer from 1 to 20. The R^2^ and mean squared error (MSE) were used to optimize the number of neurons in the hidden layer. Based on Fig. 3, the lowest value of MSE (0.0017) and the highest value of R^2^ (0.9678) were found at the number of ten neurons in the hidden layer. Hence, ten neurons in the hidden layer have the most power for the prediction of removal efficiency.

As shown in Fig. 4, the neural network architecture with 4:10:1 topology was the most appropriate ANN model. Table 1 gives the predicted value of Pb (II) ions removal efficiency by the ANN model.

According to Fig. 4, R^2^ values for training, validation, test, and overall data were calculated as 0.9534, 0.9977, 0.9971, and 0.9678, respectively. The ANN model was initially fitted on testing data and manifested a good fitness for the training data. Then, the fitness of the model was checked separately on validation and test data, giving very close predictions to the data. The appropriate distribution of data into training, validation, and test datasets and Pb (II) ions interpretation of the dataset were investigated by the evaluation of the fitness of the ANN model on the overall dataset. The high value of R^2^ confirms that the model prediction is so close to the entire experimental data and fits efficiently. This observation highlights the noticeable power of the ANN approach for modeling the treatment process. Hence, according to the ANN model, optimal reaction conditions were 5.4 pH, 1 g/L adsorbent, 24.4 mg/L concentration of Pb (II), and 71.4 min of the reaction time, for a maximum efficiency of 95.2%.

Further, the comparison of the maximum adsorption capacity of the adsorbent used in this study and other studies is shown in Table 4.

**Table 4:** Comparison of maximum adsorption capacity of Pb(II) between various adsorbents.

	Adsorbent	pH	Isotherm	Kinetic	q_m_ (mg/g)	Ref
1	Kaolinite	6	Langmuir–Freundlich	PSO	11.50	^49^
2	Montmorillonite	6	Freundlich	PSO	31.1	^49^
3	Magnetized AC	–	Freundlich	PSO	253.2	^64^
4	Tunisian Smectite	6	Langmuir	–	75.35	^65^
5	PPy/o-MWCNT	–	–	PSO	26.52	^66^
6	Magnetic bentonite	5	Langmuir	PSO–IPD	80.40	^57^
7	Modified/nanozeolites Modified (MSWI)	–	Langmuir	PSO	33	^58^
8	Syrian natural zeolite	6	Langmuir	–	33.89	^67^
9	AEAPS	6	Langmuir	PSO	27.65	^59^
**10**	**MMT-K**_**10**_	**5**	**Langmuir**	**PSO**	**40.86**	**This study**

Significant values are in bold.

## Conclusion

Pollutants like Pb (II) ions can have negative impacts on both the environment and human health. XRD, XRF, BET, FESEM, EDAX, and FTIR were employed in this study to evaluate the effectiveness of MMT-K10, which was used to remove Pb (II) ions from aqueous solutions. The effects of pH, initial concentration, contact time, adsorbent dosage, adsorption isotherm and kinetic, and optimal conditions for maximum removal efficiency were investigated, as well. The results of optimal reaction which were obtained by an artificial neural network (ANN) and genetic algorithm (GA), were reasonably close to those of RSM. The RSM determined that for a maximum efficiency of 95.149%, the ideal reaction parameters were 5.45 pH, 0.98 g/L adsorbent, 25 mg/L Pb (II), and 68 min of reaction time. Among the four studied isotherms in this work, the Langmuir model was described to be the best fit for the experimental data with R^2^ value of 0.984. The PSO model assumption was supported by the adsorption kinetic data for Pb (II) ions using MMT-K10.and the maximum sorption capacity was 2.796 mg/g at pH 5. The high adsorption capacity of MMT-K_10_ compared to other adsorbents such as Coconut coir pith activated carbon (22.8 mg/g)^53^, Ukrainian chamotte clay (11 mg/g), and Kazakhstani natural zeolite (14 mg/g)^54^ presents a good potential of this nanoclay for Pb (II) ions removal. According to these findings, it can be assured that the suggested quadratic model was appropriate for the optimization of Pb (II) removal using MMT-K_10_ and can be applied in future studies.

## Supplementary Information

Supplementary Information.

## Data Availability

All data generated or analyzed during this study are included in this published article.
