# Supplementary material for: Modeling, optimization and efficient use of MMT K10 nanoclay for Pb (II) removal using RSM, ANN and GA
Source: Sci Rep. 2023 May 24;13:8434. doi: 10.1038/s41598-023-35709-0 (PMC10209060; doi:10.1038/s41598-023-35709-0)
Supplement: Supplementary file 1 — Supplementary Information. [file 41598_2023_35709_MOESM1_ESM.docx]

**Supporting Information**

Modeling, optimization and Efficient use of MMT K_10_ nanoclay for Pb (Ⅱ) removal using RSM, ANN and GA

Farshad Hamidi^1^, Abbas Norouzian baghani^2^, Mahboobeh Kasraee^2^ , Mehdi Salari^3^, Mohammad Hadi Mehdinejad *^1^

1. *Environmental Health Research Center, Department of Environmental Health Engineering, School of Public Health, Golestan University of Medical Sciences, Gorgan, Iran*
2. *Department of Environmental Health Engineering, School of Public Health, Tehran University of Medical Sciences, Tehran, Iran*
3. *Department of Environmental Health Engineering, School of Public Health, Sabzevar University of Medical Sciences, Sabzevar, Iran*

**Corresponding author: Mohammad Hadi Mehdinejad; Email addresses:* [*hmnejad@yahoo.com*](mailto:hmnejad@yahoo.com) *Tel.: +989111718246*

*Address: Environmental Health Research Center, Department of Environmental Health Engineering, School of Public Health, Golestan University of Medical Sciences, Gorgan, Iran*

**Table of Contents**

**Table S1**. Independent variables and their levels for removal of Pb (Ⅱ) ions by MMT-K_10_.

**Table S2.** The isotherm and kinetic equations used for adsorption of Pb (Ⅱ) ions onto MMT-K_10_.

**Table S3.** BET experiment for chemical properties of the MMT-K_10_.

**Table S4.** The main chemical components of MMT-K_10_.

**Table S5.** Data for all assessed model for removal of Pb (Ⅱ) ions by MMT-K_10_.

**Fig. S1.** XRD pattern of MMT-K_10_.

**Fig. S2.** Elemental analysis of MMT-K_10_ using EDS.

**Fig. S3.** FTIR Spectra of MMT-K_10_.

**Fig. S4**. Confirming the quadratic mathematical model by predicted vs. actual (a) and residuals vs. predicted (b) for Pb (Ⅱ) ions removal in experimental runs.

**Fig. S5.** The non-linear isotherm (a) and non-linear kinetic models (b) of Pb (Ⅱ) ions removal using MMT-K_10_.

**Table S1**. Independent variables and their levels for removal of Pb (Ⅱ) ions by MMT-K_10_.

| Variable | Symbol | -1 | 0 | +1 |
| --- | --- | --- | --- | --- |
| pH | X_1_ | 3 | 5 | 7 |
| Adsorbent dosage (g/L) | X_2_ | 0.1 | 0.55 | 1 |
| Contact time (min) | X_3_ | 30 | 105 | 180 |
| Pb (Ⅱ) ions concentration (mg/L) | X_4_ | 10 | 30 | 50 |

**Table S2.** The isotherm and kinetic equations used for adsorption of Pb (Ⅱ) ions onto MMT-K_10_.

| Isotherm equations | Eq. | Kinetic models | Eq. | Removal efficiency and equilibrium adsorption capacity | Eq. | Ref. |
| --- | --- | --- | --- | --- | --- | --- |
| Freundlich |  | Pseudo-first-order (PFO) |  | Equilibrium adsorption capacity |  |  |
| $\ln q_{e}=lnk_{f}+\frac{1}{n}\ln c_{e}$ | (1) | $\ln\left( q_{e}-q_{t} \right)=lnq_{e}-k_{1}t$ | (5) | $q_{e}=\left( C_{0}-C_{e} \right)\frac{v}{m}$ | (8) | [[18-24](#_ENREF_18)] |
| Langmuir |  | Pseudo-second-order (PSO) |  | Removal efficiency |  |  |
| $\frac{C_{e}}{q_{e}}=\frac{1}{k_{1}q_{m}}+\frac{C_{e}}{q_{m}}$ | (2) | $\frac{1}{q_{t}}=\frac{1}{k_{2}q_{e}^{2}}+\frac{t}{q_{e}}$ | (6) | $R\% = \frac{C_{i}-C_{0}}{C_{0}}\times100$ | (9) | [[23-26](#_ENREF_23)] |
| Temkin |  | Intraparticle diffusion |  | Separation factor (RL) |  |  |
| qe = Bl Ln Ce + Bl Ln KT | (3) | $q_{t} = k_{i}t^{0/5} + C$ | (7) | $R_{L}=\frac{1}{(1+K_{L}C_{0})}$ | (10) | [[27](#_ENREF_27)] |
| Dubinin-Radushkevich |  |  |  |  |  |  |
| $q_{e}=Q_{m}\exp\left(-K_{D-R}\varepsilon^{2}\right)$ | (4) |  |  |  |  |  |
| q_e_ (mg/g); K_1_ (1/min); K_2_ (g/mg.min); q_m_ (mg/g); K_F_ [(mg/g) (mg/L) n] K_p_ (mg/g min^-0.5^); C_0_ (mg/L); T (K); R (8.314 J/mol.K);V (mL); m (mg) | | | | | | |

**Table S3.** BET experiment for chemical properties of the MMT-K_10_.

| Parameters | Unit |
| --- | --- |
| Density | 300-370 kg m^-3^ |
| Size of particles | 1-2nm |
| Specific surface area | 220-270m2 gr^-1^ |
| Electrical conductivity | 25mv |
| Coefficient of ion exchange | 48meq/100gr |
| Empty space between particles | 60 A^0^ |
| Color | Yellow |
| Moisture | 1-2% |

**Table S4.** The main chemical components of MMT-K_10_.

| Symbol | % Components |
| --- | --- |
| Na_2_O | 0.98 |
| MgO | 3.29 |
| Al_2_O_3_ | 19.6 |
| SiO_2_ | 50.95 |
| K_2_O | 0.86 |
| CaO | 1.97 |
| TiO_2_ | 0.62 |
| Fe_2_O_3_ | 5.62 |
| LOI | 15.45 |

**Table S5.** Data for all assessed model for removal of Pb (Ⅱ) ions by MMT-K_10_.

| Source | Sequential  p-value | Lack of Fit p-value | Adjusted R² | | Predicted R² |  |
| --- | --- | --- | --- | --- | --- | --- |
| Linear | 0.0377 | 0.0005 | | 0.2322 | 0.0543 |  |
| 2FI | 1.0000 | 0.0003 | | -0.0356 | -0.8000 |  |
| Quadratic | < 0.0001 | 0.2426 | | 0.9903 | 0.9751 | Suggested |
| Cubic | 0.1451 | 0.4262 | | 0.9953 | 0.9442 |  |

**Fig. S1.** XRD pattern of MMT-K_10_.


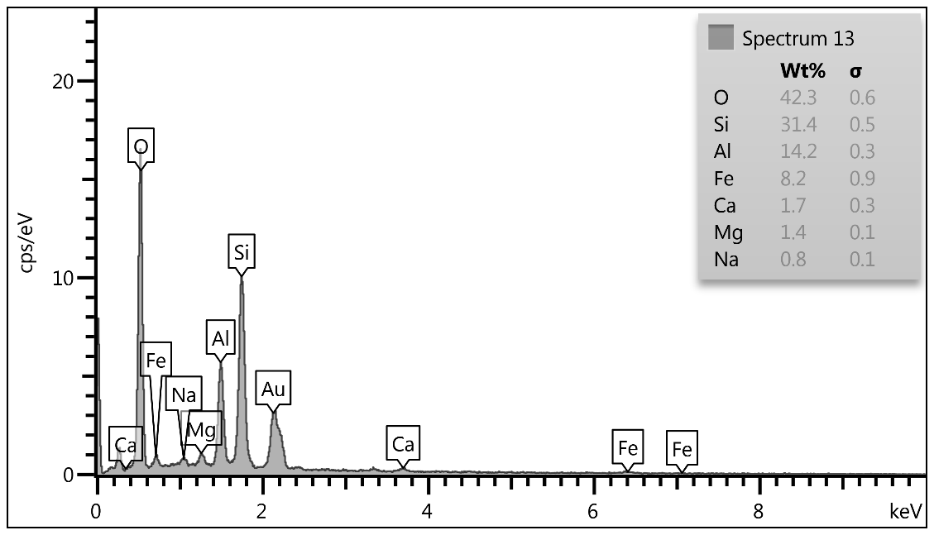


**Fig. S2.** Elemental analysis of MMT-K_10_ using EDS.

**Fig. S3.** FTIR Spectra of MMT-K_10_.


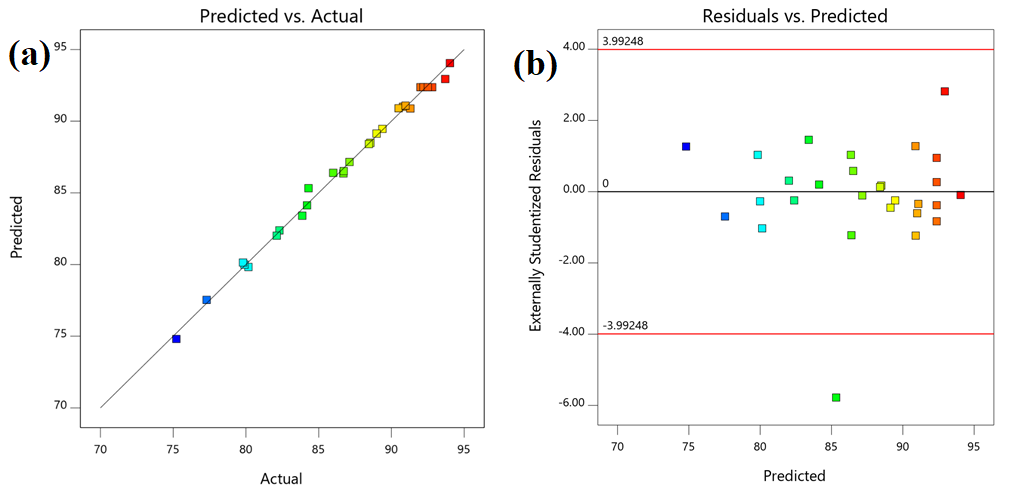


**Fig. S4**. Confirming the quadratic mathematical model by predicted vs. actual (a) and residuals vs. predicted (b) for Pb (Ⅱ) ions removal in experimental runs.

**Fig. S5.** The non-linear isotherm (a) and non-linear kinetic models (b) of Pb (Ⅱ) ions removal using MMT-K_10_.
